# Corpus callosum abnormalities, intellectual disability, speech impairment, and autism in patients with haploinsufficiency of *ARID1B*

**DOI:** 10.1111/j.1399-0004.2011.01755.x

**Published:** 2012-09

**Authors:** C Halgren, S Kjaergaard, M Bak, C Hansen, Z El-Schich, CM Anderson, KF Henriksen, H Hjalgrim, M Kirchhoff, EK Bijlsma, M Nielsen, NS den Hollander, CAL Ruivenkamp, B Isidor, C Le Caignec, R Zannolli, M Mucciolo, A Renieri, F Mari, B-M Anderlid, J Andrieux, A Dieux, N Tommerup, I Bache

**Affiliations:** a Wilhelm Johannsen Centre for Functional Genome Research, Department of Cellular and Molecular Medicine, Faculty of Health Sciences, University of CopenhagenCopenhagen, Denmark; b Department of Clinical Genetics, University Hospital of CopenhagenRigshospitalet, Copenhagen, Denmark; c Klinik for BørnCopenhagen, Denmark; d Department of Clinical Genetics, Leiden University Medical CenterLeiden, The Netherlands; e CHU Nantes, Service de Génétique MédicaleNantes, France; f Department of Pediatrics, University of SienaSiena, Italy; g Medical Genetics, Department of Biotechnology, University of SienaSiena, Italy; h Department of Clinical Genetics, Karolinska University HospitalStockholm, Sweden; i Institut de Génétique Médicale, Hopital Jeanne de FlandreCHRU de Lille, Lille, France; j Clinique de Génétique MédicaleCHRU de Lille, Lille, France

**Keywords:** *ARID1B*, autism spectrum disorder, chromosome 6q25, corpus callosum, intellectual disability, next-generation mate-pair sequencing, speech impairment, translocation

## Abstract

Corpus callosum abnormalities are common brain malformations with a wide clinical spectrum ranging from severe intellectual disability to normal cognitive function. The etiology is expected to be genetic in as much as 30–50% of the cases, but the underlying genetic cause remains unknown in the majority of cases. By next-generation mate-pair sequencing we mapped the chromosomal breakpoints of a patient with a *de novo* balanced translocation, t(1;6)(p31;q25), agenesis of corpus callosum (CC), intellectual disability, severe speech impairment, and autism. The chromosome 6 breakpoint truncated *ARID1B* which was also truncated in a recently published translocation patient with a similar phenotype. Quantitative polymerase chain reaction (Q-PCR) data showed that a primer set proximal to the translocation showed increased expression of *ARID1B*, whereas primer sets spanning or distal to the translocation showed decreased expression in the patient relative to a non-related control set. Phenotype–genotype comparison of the translocation patient to seven unpublished patients with various sized deletions encompassing *ARID1B* confirms that haploinsufficiency of *ARID1B* is associated with CC abnormalities, intellectual disability, severe speech impairment, and autism. Our findings emphasize that *ARID1B* is important in human brain development and function in general, and in the development of CC and in speech development in particular.

The corpus callosum (CC) is the main interhemispheric commissure transferring cognitive, sensory, and motor information between the two brain hemispheres. CC abnormalities include complete agenesis, hypoplasia, and varied degrees of partial agenesis (1). Agenesis of CC (ACC) occurred in 1 in 1000 in a series of unselected neonates (2) and is thus one of the most common brain malformations. It is a heterogeneous condition with a wide clinical spectrum ranging from severe intellectual disability to normal cognitive function (3, 4). The etiology is believed to be genetic in 30–50% of the cases (5, 6) whereas fetal infections and exposure to teratogenes, e.g. alcohol, are suspected causes in the remaining cases. Numerous chromosomal loci have been associated with ACC (7, 8) including loci at 6q25-q27 (8–11), but the underlying genetic cause remains unknown in the majority of cases.

Here we report eight previously unpublished patients with haploinsufficiency of *ARID1B*: one patient with a *de novo* translocation t(1;6)(p31;q25) mapped by next-generation sequencing (NGS) and seven patients with various sized *de novo* deletions.

## Materials and methods

### Patients

Each patient was clinically and molecularly evaluated by at least one of the authors. Patient 1 was identified through a national study of carriers of structural rearrangements; the study was approved by the Danish Scientific Ethics Committee and the Danish Data Protection Agency and written informed consent was obtained. Patients 2–8 were referred to genetic evaluation due to developmental delay; informed consent was obtained at the local clinical genetics departments. Patients 3–8 were identified in DECIPHER (12).

### Chromosome analysis

Standard G-banding chromosome analysis was performed on cultured peripheral lymphocytes.

### Next-generation paired-end sequencing

Mate-pair libraries were prepared using the Mate Pair Library v2 kit (Illumina, San Diego, CA). Briefly, 10 µg genomic DNA was sheared using a Nebulizer. Fragments of 2–3 kb were isolated, end-repaired using a mix of natural and biotinylated dNTPs, blunt-end ligated using circularization ligase, and fragmented to 200–400 bp. Biotinylated fragments were isolated and end-repaired and A-overhangs were added to the 3′ ends. Paired-end adapters were ligated to the fragments and the library was amplified by 18 cycles of PCR. Mate-pair libraries were subjected to 2 × 36 bases paired-end sequencing on a Genome Analyzer IIx (Illumina), following the manufacturers protocol. Reads were aligned to a reference genome using Bowtie (13) allowing up to two mismatches in the seed region. Reads not aligning uniquely were discarded from further analysis. Paired reads aligning to different chromosomes or with unexpected strand orientation were extracted to identify potential translocation and inversion breakpoints, respectively. Breakpoints were only considered as candidates if they were confirmed by at least three independent paired reads with end-reads mapping within a 6 kb region. Predicted breakpoints were filtered against known in-house variants based on data from 30 individuals with known breakpoints. Breakpoints were confirmed by PCR amplification and Sanger sequencing of the breakpoint-spanning fragments.

### Quantitative polymerase chain reaction

RNA from patient 1 and five controls was extracted from peripheral blood using standard procedures. Following extraction, RNA was DNAse I (Invitrogen, San Diego, CA) treated and reverse transcribed with a HT11V primer using SuperscriptII (Invitrogen). Primers for *ARID1B* were designed using oligo software (Molecular Biology Insights Inc., W. Cascade, CO) (Table S1, Supporting information). All primer sets were designed to span at least one intron. Q-PCR was performed on an Opticon3 thermocycler (Bio-Rad Laboratories, Hercules, CA). All samples were run in triplicates. Normalization of expression was done using two stable housekeeping genes (*EIF6* and *G6PD*). Assessment of stable housekeeping genes was done using Genorm software (14).

### Microarray analysis

Patient 1 was examined with Affymetrix Genome-Wide Human SNP Array 6.0 (Affymetrix, Santa Clara, CA). copy number variations (CNVs) >1 kb and detected by at least eight markers were identified using the Genotyping Console software (Affymetrix) and compared with variants reported in the Database of Genomic Variants.

Patient 2 was examined with Agilent Oligoarray 400K, patient 3 with Affymetrix 250K SNP array, patients 4 and 8 with Agilent 44K, patient 5 with Affymetrix 250K and Illumina Sentrix HumanHap300, patient 6 with Agilent Human Genome CGH Microarray 44 B, and patient 7 with Agilent Oligoarray 244K.

## Results

All position coordinates given below are based on Human Feb. 2009 (GRCh37/19) assembly.

### Clinical reports

Clinical data are provided in Table 1. Full clinical reports are provided in Appendix S1, Supporting information.

**Table 1:** Clinical and molecular characterization of patients with *ARID1B* haploinsufficiency^a^

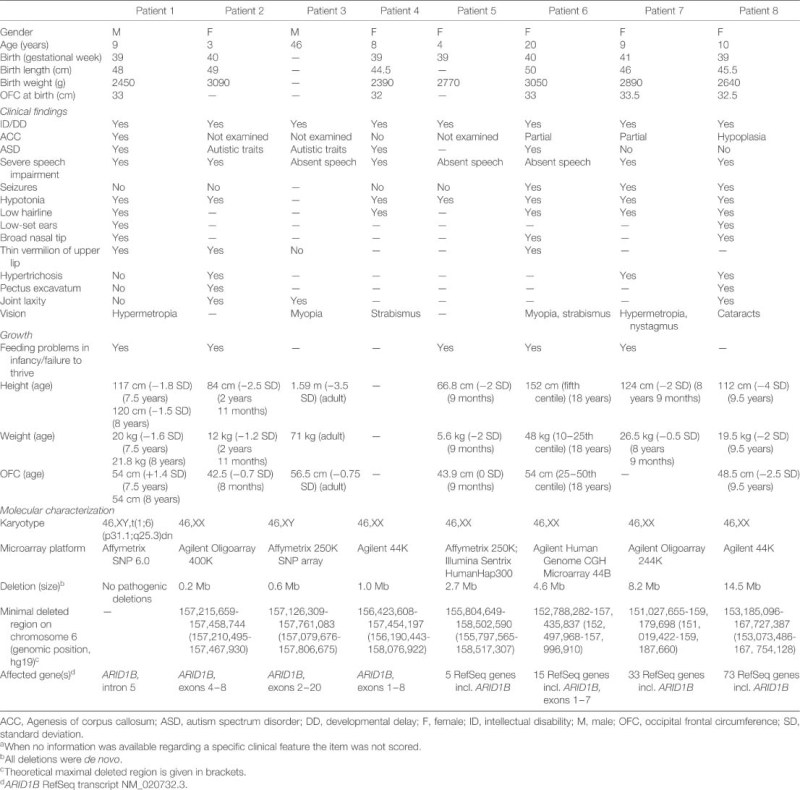

#### Patient 1

Patient 1 is an 8-year-old male. He was the first child of healthy unrelated parents. Routine second trimester ultrasound examination showed enlarged cerebral ventricles; amniocentesis was performed and a *de novo* balanced reciprocal translocation t(1;6)(p31;q25) was detected. The patient was born at term with a birth weight of 2450 g, birth length of 48 cm, and occipital frontal circumference (OFC) of 33 cm. He was hypotonic and had mild dysmorphic features. Developmental milestones were significantly delayed; he sat at the age of 2, walked at 21 /2, and spoke one to two words at the age of 3. Feeding problems and severe constipation were prominent from 6 months until 3 years of age. At the latest clinical examination, 8 years old, height was 120 cm, weight 21.8 kg, and OFC 54 cm. Dysmorphic features included a small triangular face, low hairline, micrognathia, small pointed chin, low-set large ears, broad nasal bridge and tip, concave curved thin vermilion of the upper lip, camptodactyly, and deep longitudinal plantar creases between first and second toes. He spoke only few words. Magnetic resonance imaging (MRI) showed complete ACC, and he was diagnosed with intellectual disability and autism according to the Global Assessment of Psychosocial Disability (GAPD) and the Autism Developmental Observation Schedule 1 (ADOS-1).

#### Patients 2–8

All seven patients had intellectual disability and speech impairment. Brain MRI was performed in four patients; partial ACC was detected in two of these, one had CC hypoplasia, while one patient had normal CC. Two of the patients had autism spectrum disorder (ASD) and autistic traits were found in another two. Hypotonia was reported in three patients, and four patients had feeding problems or failure to thrive in infancy.

### Chromosome analysis

**Fig. 1:**
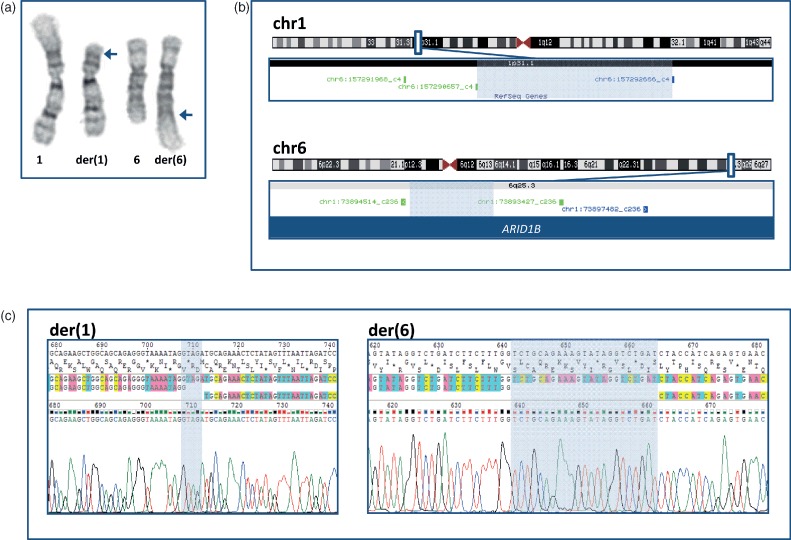
Cytogenetic and molecular characterization of patient 1. (**a**) Partial karyotype, showing a *de novo* balanced reciprocal translocation involving chromosomes 1 and 6. Cytogenetic karyotype: 46,XY,t(1;6)(p31;q25)dn. Blue arrows indicate the cytogenetically determined breakpoints. (**b**) The translocation breakpoints were mapped using next-generation mate-pair sequencing. The chromosome 1 breakpoint mapped within a 3 kb non-genic region at 1p31.1 (shaded area) and the chromosome 6 breakpoint mapped within a 700 bp genomic region at 6q25.3 (shaded area), truncating *ARID1B*. The breakpoints were detected by three reads shown in green and blue (the colors indicate the strand orientation of the reads). (**c**) By Sanger sequencing, the exact genomic positions of the breakpoints were identified to chr1:73,895,566-73,895,579 and chr6:157,292,076-157,292,079 (hg19). Four base pairs (TAGA) of unknown origin were inserted at the (der)(1) breakpoint (shaded area) and 23 base pairs (TCTGCAGAAAGTATAGGTCTGAT) were inserted at the der(6) breakpoint (shaded area); 22 of these (TCTGCAGAAAGTATAGGTCTGA) match uniquely to a LINE sequence on chromosome 7.

Chromosome analysis confirmed the karyotype 46,XY, t(1;6)(p31;q25) in patient 1 (Fig. 1a).

### Next-generation paired-end sequencing

A total of 50,180,447 paired reads were generated in a single sequencing lane. Among these, 36,082,376 paired reads passed the chastity filter, 18,515,697 paired reads aligned uniquely, and 597,838 were chimeric pairs (end-reads mapping to different chromosomes). We removed non-clustering chimeric pairs leaving a total of 311 predicted breakpoints genome-wide that were visually filtered against known variants. The translocation breakpoints, resolved from three reads, were identified at 1q31.1 and 6q25.3 (Fig. 1b), leading to the refined karyotype 46,XY,t(1;6)(p31.1;q25.3). The breakpoint at 6q25.3 truncated intron 5 of *ARID1B* (RefSeq transcript NM_020732.3) while the chromosome 1 breakpoint affected no genes. Sanger sequencing identified the exact genomic positions of the breakpoints at chr1:73,895,566–73,895,579 and chr6:157,292,076–157,292,079. Four base pairs (TAGA) of unknown origin were inserted at the chromosome 1 breakpoint, while 23 base pairs (TCTGCAG AAAGTATAGGTCTGAT) were inserted at the chromosome 6 breakpoint; 22 of these (TCTGCAGAAAGT ATAGGTCTGA) match uniquely to a LINE sequence at chromosome 7 (Fig. 1c).

### Quantitative polymerase chain reaction

**Fig. 2:**
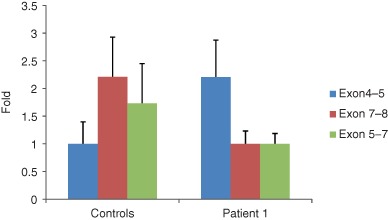
Expression pattern for *ARID1B* in patient 1 compared to five controls. Expressional levels in patient 1 using primers downstream of the translocation site were roughly half of that in the five controls. Expression levels in patient 1 using a primer set spanning the translocation likewise showed that expression was halved. Expression data obtained with a primer set located upstream of the translocation showed that the expression in patient 1 was roughly twice that of the average of the controls.

Expression of *ARID1B* was observed in all analyzed subjects. The control samples were averaged, and the expressional levels in patient 1 using primers downstream of the translocation site and spanning the translocation were roughly half compared to the controls (Fig. 2). Expression data obtained with a primer set located upstream of the translocation showed that the expression in patient 1 was roughly twice compared to the controls.

### Microarray analysis

**Fig. 3:**
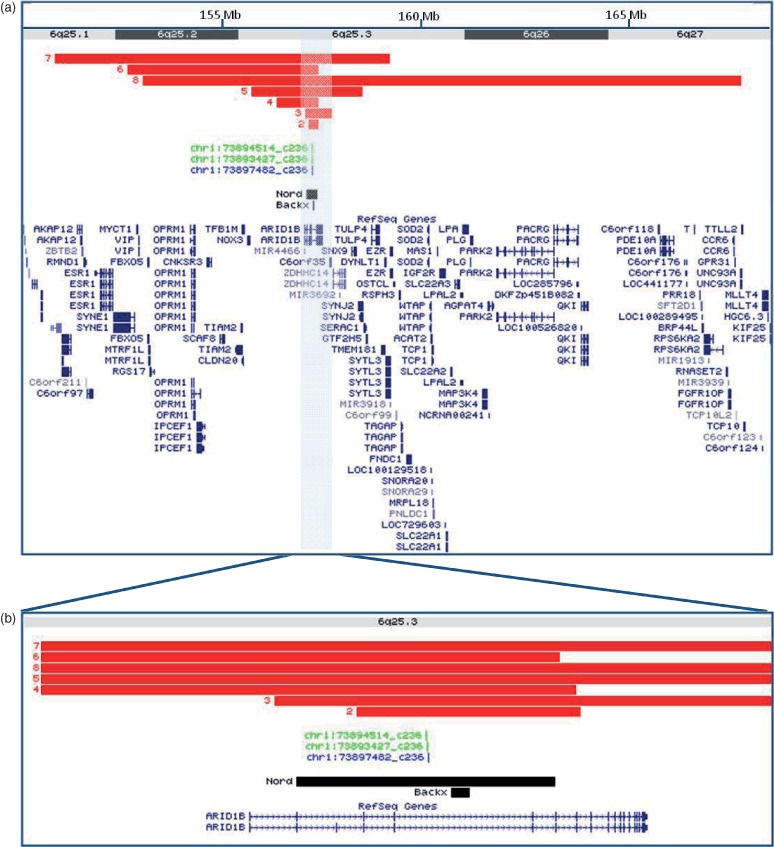
Detailed view of breakpoints and interstitial deletions affecting *ARID1B*. (**a**) Positions of interstitial *de novo* deletions affecting *ARID1B* in patients 2–8 (red bars). Green and blue ‘chr1’ reads illustrate the chromosome 6 breakpoint in patient 1. Black bars show the breakpoint previously published by Backx et al. and an intragenic deletion published by Nord et al. (**b**) *ARID1B* is disrupted in all eight patients. The figure was drawn according to the UCSC Genome Browser on Human Feb. 2009 (GRCh37/hg19) Assembly.

No potentially pathogenic CNVs were detected in patient 1. In patient 2, a 0.2-Mb intragenic deletion in *ARID1B* was detected. Deletions in patients 3 and 4 only involved *ARID1B* while patients 5–8 all had larger deletions involving 5–73 RefSeq genes. Detailed information is provided in Fig. 3 and Table 1.

## Discussion

Using NGS we showed that *ARID1B* at 6q25.3 was truncated in a patient carrying a *de novo* balanced translocation t(1;6)(p31.1;q25.3). The patient had ACC, intellectual disability, speech impairment, ASD, and mild dysmorphic features. To delineate the clinical features associated with haploinsufficiency of *ARID1B*, we compared the translocation patient to seven patients with overlapping interstitial *de novo* deletions.

We included all available patients in this study. Three patients had deletions that only affected *ARID1B*. Four patients had larger deletions encompassing 5–73 RefSeq genes, thus haploinsufficiency of other genes is likely to impact the observed phenotypes. Brain MRI was not performed on three of the reported patients; as this procedure would require general anesthesia it was decided against for ethical reasons. Despite these obvious limitations, overlapping clinical manifestations were present: all eight patients had intellectual disability, severe speech impairment, and various degrees of dysmorphic features. Callosal abnormalities were present in four of the five patients where brain imaging was performed. Three patients were diagnosed with ASD and another two showed autistic traits. This is in accordance with two recently published reports describing (i) a small *de novo* deletion within *ARID1B* in a patient with autism (15) and (ii) a patient with ACC, intellectual disability, speech impairment, and autism, in which a *de novo* translocation disrupted two genes: *ARID1B* and *MRPP3*(16). Patient 4 had normal brain MRI; this is not surprising as ACC associated loci are known to exhibit reduced penetrance (8, 11, 17). Interestingly, that same patient had intellectual disability, speech impairment, and ASD, suggesting that these traits might not be associated with visible structural brain abnormalities.

As *ARID1B* is disrupted by the translocation in patient 1, the expression of the gene could be expected to be halved unless compensatory expression was done from the normal chromosome. The observed expression pattern for primer sets downstream of or spanning the translocation was in concordance with these expectations. As data sets for exons 5–7 and 7–8 exhibit virtually identical relative expression levels, it can be indirectly inferred that the downstream fragment carrying *ARID1B*, translocated onto the derivative chromosome 1, is transcriptionally inactive as would be expected because this fragment carries no promoter region. The expressional pattern of exons located upstream of the translocation (exons 4–5) indicates that *ARID1B* is expressed at levels higher than for amplicons downstream of the translocation. This indicates that *ARID1B* is not only transcriptionally active on the normal chromosome but also from the fragment on der(6) which contains the intact promoter region. It is thus anticipated that the *ARID1B* fragment on der(1) is involved in the transcription of a chimeric mRNA consisting of the first five exons of *ARID1B* and an unknown part of chromosome 1. Interestingly these data are in concordance with previously published data from Backx et al. (16) who showed that a fusion transcript between *ARID1B* and *MRPP3* was upregulated twofold in a patient with t(6;14) and a similar phenotype. The reason for the upregulation of *ARID1B* on der(6) can only be speculated, but a TargetscanS analysis (UCSC Genome Browser http://genome.ucsc.edu/) showed the presence of multiple putative miRNA regulatory sites in the 3′ untranslated region of *ARID1B*. The lack of these miRNA regulatory sites on der(6) could easily be thought to loosen the expressional control of *ARID1B* potentially exerted by these putative regulatory sites, thus leading to increased expression from this allele. More work to confirm this theory, however, is needed.

Our findings emphasize that *ARID1B* is important for normal human brain development and function. *ARID1B* is a highly conserved gene which furthermore is associated with an evolutionary conserved stable gene desert, a hall mark of key developmental genes (18). It encodes a DNA-binding protein, ARID1B, that is part of the chromatin-remodeling complex SWI/SNF (19). Chromatin-remodeling complexes are involved in gene expression regulation. They act by altering the nucleosome structure which leads to changes in the chromatin structure that allows binding of transcriptional factors. *Arid1b* is expressed in the developing mouse brain (16) and studies of mouse embryonic stem cells have found Arid1b (BAF250b) to be particularly important in early development. Levels of BAF250b complexes were found to be high in undifferentiated mouse embryonic stem cells and lower during embryonic stem cell differentiation. Furthermore, BAF250b-deficient mouse embryonic stem cells were less capable of self-renewal and showed increased levels of differentiation (20, 21). Additional functional studies including a systematic search for *ARID1B* target genes may show how haploinsufficiency of *ARID1B* predispose to CC defects and to an array of cognitive defects, including severe speech defects.

## Supporting Information

The following Supporting information is available for this article:

Table S1. Primers used for Q-PCR examination of expressional levels of *ARID1B* in patient 1.

Appendix S1. Clinical reports of patients 2–8.
