# Supplementary material for: Corpus callosum abnormalities, intellectual disability, speech impairment, and autism in patients with haploinsufficiency of ARID1B
Source: Clin Genet. 2012 Sep;82(3):248–55. doi: 10.1111/j.1399-0004.2011.01755.x (PMC3464360; doi:10.1111/j.1399-0004.2011.01755.x)
Supplement: Supplementary file 1 [file cge0082-0248-SD1.doc]

**SUPPLEMENTARY INFORMATION**

**Clinical Reports of Patients 2-8**

***Patient 1.*** Patient 1 is an 8-year old male. He was the first child of a healthy South American mother and a European father and has an older healthy half brother (same father). Routine second trimester ultrasound examination revealed enlarged cerebral ventricles. Amniocentesis was performed and chromosome analysis showed an apparently balanced *de novo* reciprocal translocation 46,XY,t(1;6)(p31;q25) (Figure 1A). The patient was born at term via uncomplicated vaginal delivery. Birth weight (BW) was 2,450 g, birth length (BL) 48 cm, and occipital frontal circumference (OFC) 33 cm. He was slightly hypotonic, had poor suck, and delayed Moro reflex and mild dysmorphic features were noted. Developmental milestones were significantly delayed; he sat independently at the age of 2, walked at 2½, and spoke one or two words at the age of 3. Feeding problems and severe constipation were prominent from 6 months until 3 years of age. He had delayed bone age, bilateral cryptorchism, slight hypermetropia (+2/+2.25), and normal hearing. At the age of 7½, his height was 117 cm (-1.8 SD), weight 20 kg (-1.6 SD), and OFC 54 cm (+1.4SD). At the latest clinical examination, 8 years old, height was 120 cm (-1.5 SD), weight 21.8 kg, and OFC 54 cm. Both parents had short stature; the father was 162.5 cm (-2.7 SD) and the mother 146.5 cm (-3.6 SD). Dysmorphic features included a small triangular face, low hairline, micrognathia, small pointed chin, low-set large ears, broad nasal bridge and tip, concave curved thin vermilion of the upper lip, camptodactyly, and deep longitudinal plantar creases between 1^st^ and 2^nd^ toes. His spoken language was sparse consisting of no more than a few words. He was diagnosed with intellectual disability and autism according to the Global Assessment of Psychosocial Disability (GAPD) and Autism Developmental Observation Schedule 1 (ADOS-1). Brain MRI showed complete agenesis of corpus callosum (ACC), enlarged posterior part of lateral ventricles, and enlarged anterior part of lateral ventricles.

**Patient 2** is a 3-year old girl. She was the second child of healthy unrelated Caucasian parents. Family history was normal except for an older sister with cleft lip/palate. The patient was born by vaginal delivery at term after an uncomplicated pregnancy. BW was 3,090 g, and BL 49 cm. 8 months old, OFC was 42.5 cm (-0.7 SD). She had failure to thrive during the first year of live. She suffered from recurrent upper respiratory tract infections and asthmatic bronchitis, and had low immunoglobulins (IgA and IgG). She had development delay, e.g. walked at age 21 months, and spoke only a few single words at age 2½ years. She had an open mouth appearance, protrusion of the tongue, and increased salivation, and severe joint laxity mostly affecting the lower extremities. At the most recent examination, age 2 years and 11 months, she was found to have hypotonia and an unsteady balance. Mild dysmorphic features included thin vermilion of the upper lip, hypertrichosis of the upper back, and mild pectus excavatum. Height was 84 cm (-2.5 SD) and weight 12 kg (-1.2 SD). She was a happy girl with autistic behavior, e.g. tended to avoid other children and appeared to be in a world of her own. No brain imaging was performed.

**Patient 3** is a 46 year-old male with severe developmental delay, autistic behavior, absent speech, anal atresia, and double ureters. At the latest clinical examination height was 1.59 m (-3.5 SD), weight 71 kg, and OFC 56.5 cm (-0.75 SD). Dysmorphic features included an asymmetrical and coarse face with a prominent nose, dysplastic ears, thick vermilion of the lips, and large tongue. HHe had temporal balding, a thoracic kyphosis, broad distal phalanxes of hands and feet, and joint laxity. He had a slight hearing loss (40 db bilaterally) of unknown cause and severe myopia (-9 bilaterally). The patient displayed repetitive behavior and pulling of toe nails but did not otherwise show self mutilating behavior. No brain imaging was performed. An electroencephalogram (EEG) was normal.

**Patient 4** is a 9-year old girl. She was the first child of healthy unrelated parents. She was born by uncomplicated vaginal delivery at term with a BW of 2,390 g, BL 44.5 cm, and OFC 32 cm. Hypotonia was noticed in the neonatal period. She had global developmental delay, e.g. sat at 9 months, walked at 2 years, and spoke single words at 3 years. At age 6 she was diagnosed with intellectual disability and ASD according to ADOS and the Autism Diagnostic Interview (ADI). Dysmorphic features included a low frontal hairline, thick eyebrows, strabismus, a large nose, and short hands. MRI of the brain performed at age 4 years and 5 months was normal.

**Patient 5** is a 4-year old girl. She was the first child of healthy unrelated Caucasian parents. She was born by uncomplicated vaginal delivery at term with a BW of 2,770 g. The neonatal period was complicated by feeding difficulties and growth retardation and she received tube feeding for several months during the first year of life. At 9 months her height was 66.8 cm (-2 SD), weight 5.6 kg (-2 SD), and OFC 43.9 cm (0 SD). Developmental milestones were severely delayed; e.g. head lag at 9 months. At the age of 10 months she underwent surgery to close an atrial septum defect and remove a bronchogenic cyst. Dysmorphic features included plagiocephaly, broad forehead, deeply set eyes, upslanted palpebral fissures, long philtrum, a wide mouth, and a large tongue. She had a single palmar crease on the left hand and dysplastic nails on both fifth toes. Her hair was sparse and her nails did not grow. Four years old she developed hypercalcaemia and renal lithiasis. She had no speech. No brain imaging was performed.

**Patient 6** is a 20-year old woman. She was the third child born to healthy unrelated parents. Pregnancy was complicated by oligohydramnios and risk for preterm labor. She was born by uncomplicated vaginal delivery at term with BW 3,050 g, BL 50 cm, and OFC 33 cm. Neonatally generalized hypotonia, hypothermia, feeding difficulties, and mild dysmorphic features were noted. Developmental milestones were delayed, e.g. walked at 30 months of age. Since early childhood she suffered from complex partial seizures; EEG showed paroxysmal activity in the left temporal region and right frontal region. She was diagnosed with intellectual disability and ASD. At the latest clinical examination, 18 years old, height was 152 cm (5^th^ centile), weight 48 kg (10-25^th^ centile), and OFC 54 cm (25-50^th^ centile). Dysmorphic features included a long face with premature aging, low frontal hairline, hypotelorism, a broad nasal tip, thin vermilion of the upper lip, and thick everted vermilion of the lower lip. She had no speech. She had strabismus, severe myopia, flat arches of feet, asymmetrical breasts, supernumerary nipple, and winged scapulae. Brain MRI showed mega cisterna magna, agenesis of splenium and the posterior portion of CC.

**Patient 7** is a 9-year old girl. She was the first of two children born to unrelated healthy parents; her younger brother was healthy. She was born at term by vaginal delivery after a normal pregnancy with BW 2,890 g, BL 46 cm, and OFC 33.5 cm. Paleness, difficulties in breast feeding, bradycardia, and heart murmur were noticed during the neonatal period. Developmental milestones were delayed, e.g. she walked at 2 years and spoke single words at 3 years. When 4 years old, she presented with absences seizures and an EEG showed epileptiform activity with solitary and joined sharp-wave complex within the left hemisphere with a maximum centrotemporal. Dysmorphic features included deeply set eyes, downslanted palpebral fissures, low frontal hairline, wide mouth, thick vermilion of the lower lip, microdontia. Fingers and toes were broad with sandal gap. Hypertrichosis was noticed on arms and back. She had nystagmus, the vision was impaired, and the hearing was normal. At the age of 8 years and 9 months her height was 124 cm (-2 SD) and weight 26.5 kg (-0.5 SD). She had problems with concentration and focus, severe expressive language disorder (said only yes or no), and there was no eye contact. Brain MRI showed partial ACC, slightly smaller splenium CC, thin anterior commisure, thin tracti optici.

**Patient 8** is a 10-year old girl. She was the first child to healthy unrelated Caucasian parents. Prenatal ultrasound examination revealed increased nuchal translucency and a chromosome analysis was performed. She was born by uncomplicated vaginal delivery at term with BW of 2,640 g, BL 45.5 cm, and OFC 32.5 cm. The child was hypotonic, and webbed neck and a right preauricular pit were noticed. Developmental milestones were markedly delayed; e.g. she walked independently at 4 years of age. She suffered from renal lithiasis from the age of 4, and she developed bilateral cataract at the age of 6 years. She suffered from partial seizure epilepsy. At the most recent examination at age 9½ years she had microcephaly, hyperactivity, and speech impairment (spoke only 2-3 words). Dysmorphic features included a very low posterior hair line, low set posteriorly rotated ears, deviated nasal septum, broad nasal tip, and a long flat philtrum. The mouth was frequently open with a protruding tongue. She had slender fingers, joint contractures of the knees, general joint laxity, pectus excavatum, and hypertrichosis. Height was 112 cm (-4 SD), weight 19.5 kg (-2 SD), and OFC 48.5 cm (-2.5 SD). Brain MRI showed hypoplasia of CC, moderate vermis hypoplasia, large ventricles.
