# Supplementary material for: Corpus callosum abnormalities, intellectual disability, speech impairment, and autism in patients with haploinsufficiency of ARID1B
Source: Clin Genet. 2012 Sep;82(3):248–55. doi: 10.1111/j.1399-0004.2011.01755.x (PMC3464360; doi:10.1111/j.1399-0004.2011.01755.x)
Supplement: Supplementary file 2 [file cge0082-0248-SD2.doc]

**Supplementary Table**

**Primers used for Q-PCR examination of expressional levels of *ARID1B* in patient 1.**

| Primers | Exon | Location relative to translocation | Annealing Temp. |
| --- | --- | --- | --- |
| TCAGTATGGACAGCAAGGTGTGAGTGGTT GGGGGCCAGAGGAGGTTGAGATCT | 4-5 | Upstream | 63 |
| GAATCCAGTTCCCATCCCGCCTTGA  CGCTGGGGGTCTGGAGTAGTTACCTTGTG | 5-7 | Spanning | 63 |
| CAGATCTGTCTGGCTCCATTGATGACCTC  AACCTCTTTCCTGTGGCATTGGTGACTG | 7-8 | Downstream | 63 |
| AAGTCTTCAGACAGACAGTGGCCGACCAG  ACCACCATCCCAGCAGCAATCACCT |  | EIF6 | 64 |
| CCGGGACAACATCGCCTGCGTTATC  ACGGCTGCAAAAGTGGCGGTGGT |  | G6PD | 64 |
